# Oncology Camp Participation and Psychosocial Health in Children Who Have Lived with Cancer—A Pilot Study

**DOI:** 10.3390/curroncol31110528

**Published:** 2024-11-15

**Authors:** Sarah O’Connell, Nathan O’Keeffe, Greg D. Wells, Sarah L. West

**Affiliations:** 1 Department of Biology, Trent University, Peterborough, ON K9L 0G2, Canada; 2 Translational Medicine, The Hospital for Sick Children, Toronto, ON M5G 1E8, Canada; 3 Department of Kinesiology, Trent University, Peterborough, ON K9L 0G2, Canada

**Keywords:** cancer, camp, psychosocial health, resilience, hope, children, youth

## Abstract

Children with lived cancer experience encounter adversity, therefore experiences promoting psychosocial health are necessary. This pilot study determined the impact of recreational oncology camps (ROC) on resilience, hope, social support, and mental well-being in youth who have lived with cancer. Youth (6–18 years) with cancer experience enrolled in an 11-day session of ROC (Muskoka, Ontario, Canada) were invited to participate. Participants completed a survey [Children’s Hope Scale (CHS), Child and Youth Resilience Measure (CYRM-R), Social Provisions Scale (SPS-5), and Short Warwick–Edinburgh Mental Wellbeing Scale (SWEMWBS)] on the first (T1) and last day (T2) of camp, and 3 months post-camp (T3). Repeated-measures ANOVAs evaluated differences in survey scores among time points. Ten participants (14.1 ± 2.5 years) were included in the analysis. CHS scores at T3 were lower than T1 and T2 (F = 9.388, *p* = 0.008). CYRM-R, SPS-5, and SWEMWBS scores were high but did not differ between time points. Hope decreased 3 months post-camp, suggesting a need for continued psychosocial support. Overall, the ROC environment is associated with positive psychosocial health.

## Simple Summary

This pilot study examined the association of participating in a recreational oncology camp (ROC) with the psychosocial health (resilience, hope, social support, and mental well-being) of children aged 6 to 18 who have experienced cancer. Participating youth completed surveys at the start and end of an 11-day ROC, as well as three months later. While the results showed that children experienced strong psychosocial health during the ROC, their levels of hope decreased after three months, suggesting a need for ongoing support. This study highlights the importance of recreational programs like ROC in promoting psychosocial health among youth who have experienced cancer.

## 1. Introduction

Children and adolescents with cancer experience numerous stressors including painful procedures, significant physical changes, and treatment-related functional impairments as a result of dealing with a life-threatening disease [1,2]. As well, these children are often removed from their normal environments, routines, and activities due to treatment and associated side effects, thereby causing them to miss key opportunities to interact with peers [3]. Cancer treatment can lead to long-term physical and psychosocial complications, commonly referred to as the late effects of cancer, and children with cancer are particularly susceptible to these late effects as they undergo cancer treatment during a critical period for physical and psychosocial development [4,5,6]. Psychosocial health encompasses emotional, psychological, and social health and is determined by both low levels of negative affect and psychological symptoms (e.g., anxiety) and the presence of positive aspects of psychosocial wellbeing, like positive mood and life satisfaction [7,8]. Opportunities that promote psychosocial health and social interaction in youth who currently have or have had cancer are important, particularly because childhood is a critical period for psychosocial growth [5].

Recreational oncology camps (ROCs) are one example of a program that may target psychosocial health; they provide a medically safe and supportive environment where youth who have lived experience with cancer can engage in social interaction and take part in a traditional camp experience with peers who have similar medical experiences. Depending on the ROC, they may offer day camp or overnight camp for children and/or families affected by childhood cancer. ROCs often have medical staff onsite and can provide support to ensure the safety of children undergoing cancer treatment. Currently, there are 16 ROC organizations in Canada [9].

Qualitative studies suggest that ROCs foster a communal environment where children feel accepted by others, experience a sense of normalcy and respite from the daily challenges of cancer treatment, and develop friendships [10,11,12]. ROCs are associated with improvements in friendship skills, self-esteem, and health-related quality of life [13,14,15]. Despite being a promising opportunity to promote psychosocial health in this patient population, there are limited quantitative studies that evaluate the impact of ROCs on the psychosocial health (both positive and negative psychosocial outcomes) of youth with lived experience of cancer [14,16,17].

Therefore, the current study evaluated the association of participating in an 11-day, in-person ROC on the psychosocial health of youth with lived experience with cancer. We hypothesized that attending an 11-day in-person ROC would improve positive psychosocial indicators (hope, resilience, perceived social support, and overall mental well-being).

## 2. Materials and Methods

### 2.1. Procedure

This prospective study was approved by the Research Ethics Board at Trent University (REB #28022). Children and adolescents with lived experience of cancer who were attending an 11-day camp session in the summer of 2022 at an ROC in Ontario were recruited for this study. This camp session was exclusively for the attendance of childhood cancer patients (and not siblings and/or their families). Campers participated in various activities throughout the 11-day camp session, including waterskiing, arts and crafts, high-ropes courses, pottery, music, campfires, canoeing, kayaking, swimming, and fishing, among other activities.

Prospective participants were contacted via email from the ROC’s registration list. An email containing a letter of assent/consent and study information was sent to prospective participants and their parent(s)/caregiver(s). Eligibility criteria for the study included: (1) aged 6 to 18 years; (2) has a cancer diagnosis; (3) is at any stage of treatment, including post-treatment. Campers who provided informed assent/consent (signed by themselves and their guardian) completed study testing at three time points: baseline (day 1; upon arrival at camp), post-camp 1 (day 10; the last full day of camp), and post-camp 2 (three months after camp).

Surveys were administered at each time point via the Qualtrics survey platform. Surveys at baseline and post-camp 1 were administered with members of the research team, who sat with each participant. Post-camp 2 surveys were completed independently by participants. All survey responses were linked to each participant’s unique study ID. Each survey took approximately 10 min to complete and was the same at each time point. The following validated scales were administered at each time point: Child and Youth Resilience Measure (CYRM), Children’s Hope Scale (CHS), Social Provisions Scale-5 (SPS-5), and Short Warwick–Edinburgh Mental Wellbeing Scale (SWEMWBS) [18,19,20,21]. The survey administered at baseline testing also included demographic-related questions (self-reported age, gender, and race). Cancer-specific information, including primary cancer diagnosis, date of first cancer diagnosis, treatment status, and the number of years of camp attendance, were extracted from camp records by staff from the ROC.

### 2.2. Children’s Hope Scale

The Children’s Hope Scale (CHS) was used to assess hope [19]. The CHS is a 6-item scale that encompasses agency and pathways of thinking to assess overall levels of hope [19]. It has been validated in youth [19,22]. Responses to each item were on a 6-point Likert scale. Scores for all items were directly summed to obtain a total hope score (6 to 36), and scores for the odd-numbered and even-numbered items were directly summed to calculate the total agency and pathways scores (3 to 18), respectively. Higher scores indicate higher levels of hope through the ability to develop routes towards goals (pathways) and to initiate and sustain progression towards goals (agency) [19].

### 2.3. Child and Youth Resilience Measure

Resilience was measured using the Child and Youth Resilience Measure (CYRM) [18]. The Rasch-validated CYRM includes 17 items and has been validated in youth [18,23]. To better contextualize the CYRM, two items were removed from this measure because they were not relevant to the children’s camp experience. Item 6, “Is there enough to eat in your home when you are hungry?”, and item 4, “Do you feel that your parent(s)/caregiver(s) know where you are and what you are doing all of the time?”, were removed [23]. As such, 15 items remained on the CYRM that was administered for this study. When completing this scale, participants scored each item on a 5-point Likert scale. Scores for all items were directly summed to obtain a total score (15 to 75) with higher scores reflecting higher levels of resilience.

### 2.4. Social Provisions Scale-5

The Social Provisions Scale-5 (SPS-5) was used to measure social support. The SPS-5 is a brief version of the original SPS [24]. This scale included 5 subscales (one item per scale) and each subscale measures a different social function (provision) that can be obtained from interpersonal relationships, as described by Weiss’ (1974) model of social provisions [20,25]. The SPS-5 has been validated against the first short form of the SPS (SPS-10) [20]. Responses were based on a 4-point Likert scale and scores for all items were directly summed to obtain a total score (5 to 20). Higher scores indicated higher levels of perceived social support.

### 2.5. Short Warwick–Edinburgh Mental Wellbeing Scale

Mental well-being was determined using the Short Warwick–Edinburgh Mental Wellbeing Scale (SWEMWS). This is a 7-item, Rasch-validated scale that assesses overall mental well-being through measures of feeling good (hedonia) and functioning well (eudaimonia) [21,26,27]. Responses to items were on a 5-point Likert scale and scores were directly summed to obtain a total score (7 to 35). Higher scores indicated better overall mental well-being.

### 2.6. Statistical Analysis

Repeated-measures analysis of variance (ANOVA) tests (and Tukey’s post-hoc) were used to determine whether there were differences in hope, resilience, social support, and mental well-being between the three time points (baseline, post-camp 1, and post-camp 2). The effect size was calculated by Cohen’s d between each timepoint (0.20 = small effect size; 0.50 = medium effect size; 0.80 = large effect size) [28]. Wilcoxon signed ranks tests were performed to evaluate whether levels of each outcome differed between baseline and post-camp 1. All statistical analyses were performed using SPSS (version 29.0) statistical software, and significance was accepted at *p* < 0.05.

## 3. Results

### 3.1. Demographic Characteristics

A total of 84 youth were enrolled to participate in this camp session. Twenty-six campers and their guardians provided assent/consent to participate. Twenty-three participants completed testing at the first two time points (due to testing time constraints, and/or participants having left camp early). A total of 10 participants completed study testing at all three time points. When evaluating differences between time points one and two, overall findings did not differ between analyses including campers who participated in baseline and post-camp 1 testing (*n* = 23) versus testing at three time points (*n* = 10). As such, the 10 participants who completed study testing at all time points are presented in the current analysis. The demographic characteristics of the 10 individuals who participated in this study are summarized in Table 1. Over half of the participants identified as female, with most participants identifying as Caucasian. The primary cancer diagnosis was leukemia, and the mean number of years that participants had been attending overnight ROC was ~5 years.

### 3.2. Psychosocial Health

**Figure 1:**
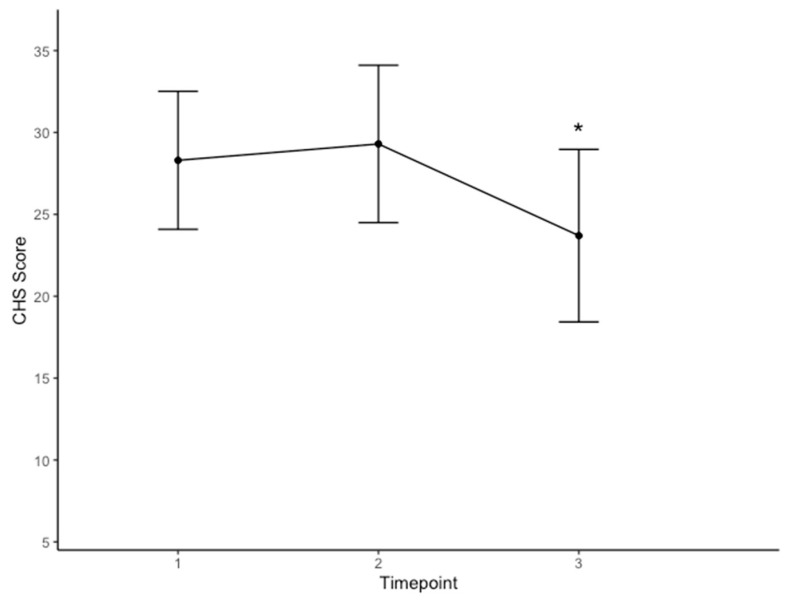
Mean total hope scores from the Children’s Hope Scale (CHS) across the three time points (1 = baseline; 2 = post-camp 1; 3 = post-camp 2). Error bars represent standard deviation (*n* = 10). * denotes a significant difference between post-camp 2 CHS scores vs. CHS scores at baseline and post-camp 1 (*p* = 0.047; *p* = 0.023).

Mean total hope CHS scores are illustrated in Figure 1 (*n* = 10). CHS scores did not change from baseline to post-camp 1; however, CHS scores at post-camp 2 were significantly lower than those at baseline and post-camp 1 among participants (F = 9.388, *p* = 0.008). Similarly, CHS scores on the agency and pathways subscales at post-camp 2 (12.30 ± 3.917; 11.40 ± 3.688) were significantly lower than those at baseline (14.40 ± 3.169; 13.70 ± 3.020) and post-camp 1 (15.00 ± 2.906; 14.30 ± 3.945) among participants (F = 5.233, *p* = 0.035; F = 9.084, *p* = 0.009).

Mean scores for all other psychosocial outcomes are reported in Table 2. There were no significant differences in scores for resilience (CYRM), social support (SPS-5), or mental well-being (SWEMWBS) between the time points (F = 2.536, *p* = 0.140; F = 1.594, *p* = 0.261; F = 3.066, *p* = 0.103, respectively). However, Cohen’s d revealed a large, moderate, and small effect size (decrease) in SPS-5, SWEMWBS, and CYRM scores, respectively, from post-camp 1 to post-camp 2 (d = 0.80; d = 0.79; d = 0.43). Furthermore, based on the provided reference ranges for these scales, mean scores on the CYRM, CHS, and SWEMWBS decreased into a lower reference range from baseline/post-camp 1 to post-camp 2. Mean scores for each scale at baseline, post-camp 1, and post-camp 2, along with their corresponding reference ranges, are summarized in Table 2.

## 4. Discussion

The current pilot study investigated the impact of an 11-day, overnight ROC experience on the psychosocial health of childhood cancer patients. To our knowledge, this was the first quantitative study to evaluate how in-person ROC programming may affect the hope, resilience, and overall mental well-being of youth with lived cancer experience. We hypothesized that the psychosocial health of childhood cancer patients would be improved by ROC attendance. While our results did not support this hypothesis, as there were no statistically significant improvements between the first and last day of camp in any of the psychosocial outcomes measured, we report that while attending ROC, children with lived experience of cancer exhibited high levels of resilience, hope, perceived social support, and overall mental well-being. Furthermore, we found that levels of hope among youth with lived experience of cancer were high while they were in the camp environment but decreased to moderate levels three months post-camp.

### 4.1. Hope

We found levels of hope to be within the high range in our study participants while attending ROC. The high levels of hope in youth with lived cancer experience is important as hope is associated with lower levels of distress and better quality of life in adolescents and young adults with cancer, as well as reduced anxiety and depression in children with cancer [31,32,33,34]. Hope is also associated with better medication adherence in children with chronic diseases, which is especially important as adolescents with cancer may not adhere to their treatment regimen in an effort to feel more “normal” [35].

Importantly, we found that levels of hope were significantly higher in youth with lived cancer experience while they were in the ROC environment compared to when they were in their regular environments three months post-camp. Decreased levels of hope three months post-camp could be attributed to multiple factors, such as changes in the severity of their illness, prognosis, or late effects, as well as other general life factors (e.g., family, friend, and/or school-related factors). For instance, one study found that lower levels of hope were associated with greater disease severity in youth with sickle cell disease [36]. As such, it is possible that if participants returned home and experienced worsened physical health, their levels of hope could have decreased.

It is also possible that participants experienced less freedom when in their regular environment versus at ROC. ROC is associated with improvements in freedom and self-efficacy, which provide individuals with a sense of control, thereby promoting hope [14,37,38]. As such, reduced levels of freedom in participants’ regular environments could have contributed to the decreased levels of hope we observed. Finally, ROC may have been a more goal-oriented environment than participants’ regular environments as campers encounter new activities and challenges at camp. These challenges may encourage youth to develop goals (agentic thinking) and through accomplishing their goals, they build their confidence and self-esteem—components of hope [11,12,39]. In addition to these challenges, ROC provides youth with opportunities to engage in social comparisons with individuals who have similar illness-related experiences [40,41]. Conversely, when youth return to their regular environments and are surrounded by individuals who may not be living with cancer, their ability to make social comparisons with an appropriate group is limited, which can lead to frustration and discourage their goal pursuits [40]. Therefore, the goal-oriented nature of the ROC environment could have promoted hopeful thinking in youth more than in their regular environments and this could have contributed to the decreased levels of hope we observed three months post-camp.

To our knowledge, this is the first study to quantify hope solely in youth with lived cancer experience during and following an in-person ROC experience. Interestingly, our hope findings were inconsistent with those of Woods et al. (2013), who reported significant improvements in levels of hope, from pre- to post-camp, among youth aged 8 to 19 years who attended a camp designed for children with chronic illnesses (including cancer) [42]. These discrepancies could be attributed to differences between the camps. It is possible that the experiences offered by a non-disease-specific camp differ in ways that affect hope differently. Additionally, differences in participant demographics may explain these discrepancies as our study evaluated children with lived cancer experience exclusively, while Woods et al. (2013) [42] evaluated youth with various chronic illnesses. Our participants also reported higher baseline hope scores compared to Woods et al. (2013) [42]; therefore, we may not have seen improvements in levels of hope because our participants already had high levels of hope. The high levels of hope we report are particularly promising as levels of hope in adolescents and young adults (aged 12 to 25 years) have previously been reported to be within the moderate range [43].

### 4.2. Resilience

While attending ROC, youth with lived cancer experience exhibited high levels of resilience (63–67 on a 15-item scale) compared with scores from healthy youth, but they only had moderate levels of resilience three months post-camp [23]. Although not statistically significant, this decrease based on scoring thresholds suggests there may be a clinically meaningful decrease in the resiliency of children with cancer after leaving the ROC environment. Clinically meaningful findings refer to treatment or intervention effects that have a real impact on individuals’ daily lives, including their quality of life, their ability to function, and/or how the treatment makes them feel [44]. We suggest that a change from high to moderate levels of resilience post-camp in the current study is a relevant finding.

Goal orientation, which is a large component of the definition of hope used in this study, is a resilience factor [45]. Our clinically meaningful decrease in resilience could be attributed to the decreased levels of hope, and presumably goal orientation, we found in youth between when they were in the ROC environment and when they were in their regular environments. Social support and connectedness (i.e., reciprocal social relationships) are resources that can be used to facilitate resilience [46]. However, it is unlikely that social support was the primary factor contributing to resilience in our participants as their high levels of social support in the ROC environment were sustained when they returned to their regular environments three months later. As such, the potentially clinically meaningful decrease in resilience three months post-camp is unlikely to be explained by changes in levels of social support.

Somewhat in contrast to our findings, Wynn et al. (2012) reported that adolescents (aged 17 to 21 years) with cancer appeared to demonstrate an improvement in 12 out of 14 aspects of resilience, using the 14-item Resilience Scale, after participating in an adventure-based program that included an eight-day wilderness journey in New Zealand [47]. Although they used a validated scale to measure resilience, they did not perform statistical analyses to determine the impact of adventure therapy on resilience. Their study was also limited by a smaller sample size (N = 5) than our current study. To our knowledge, our study is the first to have quantitatively evaluated the impact of camp programming on the resilience of youth with lived cancer experience.

### 4.3. Social Support

Our finding of high levels of perceived social support in youth with lived cancer experience attending ROC is consistent with current qualitative literature. Most studies investigating the experiences of children with cancer who attend ROC report that children develop close relationships with other campers, feel a sense of community, and/or feel that they have a social support network at ROC [10,37,48]. Furthermore, our finding that levels of social support did not change across the time points is consistent with quantitative literature investigating social support in children attending ROC [15]. Békési et al. (2011) reported that levels of social support did not change from pre- to post-camp in their pediatric cancer population attending a camp designed for children with chronic diseases [15].

It is important to provide youth who have experienced cancer with an environment that allows them to form social relationships in which they can derive social support, as social isolation is common in childhood cancer patients/survivors [49]. One study reported that 19 out of 30 of their participants who were adults who survived childhood cancer experienced social isolation at some point in their lives [49]. We found that levels of perceived social support were high while in the ROC environment, and these high levels of perceived social support were sustained three months post-camp in youth with lived cancer experience. Importantly, a 2014 qualitative study investigating the experiences of adult childhood cancer survivors who attended ROC found that many childhood cancer survivors experienced continued social support through survivorship and adulthood from the people they formed connections with at camp [10]. Therefore, the social support received at ROC may help reduce social late effects and social isolation during treatment and throughout survivorship.

### 4.4. Mental Well-Being

When compared to the available normative population values for the SWEMWBS, scores of the youth with lived cancer experience in this study were above the 75th percentile while at ROC [30]. Notably, mean SWEMWBS scores dropped below the 75th percentile three months post-camp [30]. Our findings of high levels of mental well-being in youth while attending ROC are largely consistent with the current literature. In their narrative review, Neville et al. (2019) concluded that most findings in the literature suggest that ROCs have a positive impact on the psychosocial well-being of youth with lived cancer experience [41]. Upon comparing mental well-being scores between post-camp 1 and post-camp 2, we found the effect size to be moderate (decrease). This suggests there may be a clinically meaningful change in mental well-being when youth are back in their regular environment compared to when they were at camp. It should be noted that the available population norms are based on data from adults, so comparisons should be made with caution as they may not be generalizable to a pediatric population.

From a developmental perspective, ROC may have offered participants unique experiences that could promote their overall well-being, particularly considering that most of our participants were adolescents and in a stage of development where establishing a sense of autonomy and identity is critical [35,50]. Youth with cancer commonly report frustrations with establishing their identity apart from their cancer or others’ perceptions of them as the “kid with cancer” [35,37]. Furthermore, adolescents with cancer commonly report feeling different from their healthy peers, which is problematic as peer relationships aid in the formation of identity in adolescents [40,51]. Difficulties with developing a sense of identity and/or belonging with a peer group can lead to role confusion and can result in risk-taking behaviors, like medication non-adherence, as they may attempt to regain some sense of normalcy [35]. However, adolescents attending ROC have reported feeling accepted by and similar to their camp peers [37,40,41]. ROC may also provide youth with cancer a sense of freedom and normalcy [10,37,41,48]. As such, ROC may promote the well-being of adolescents with cancer by providing them with normalizing experiences, an accepting environment in which they can explore their identity, and a sense of belonging to a peer group, all of which can reduce role confusion [37,40,41].

### 4.5. Limitations

There are several limitations to this study. First, we were unable to collect data prior to the participants arriving at camp (i.e., a baseline collection point while children/adolescents were in their usual environments). Our baseline data collection point was when participants arrived on the first day of the ROC session; most children arrived via bus, and thus they had already begun the camp experience and they may already have been experiencing excitement and other emotions, which could have affected their survey scores. However, we suggest that the survey scores we obtained from participants three months post-camp may be reflective of what participants experience in their normal environment. That said, future studies with baseline testing taking place before the start of the camp session are needed. Furthermore, survey scores were quite high at baseline; therefore, we may have encountered a ceiling effect that prevented us from observing significant improvements in measures of resilience, hope, social support, and mental well-being. Therefore, baseline testing should occur prior to the start of the summer/ROC session as this would control for confounding emotions associated with arriving at ROC, and it would allow for better comparison with the three-month follow-up as both would occur in the context of the academic year with similar environments and stressors.

This study was also limited in its ability to determine causal relationships, as we did not collect data from a comparison group of children who did not attend ROC. As such, we were only able to infer correlational relationships between ROC attendance and the psychosocial outcomes of interest. Future studies may include a control group, such as siblings attending ROC, to allow for further comparisons.

Our relatively small sample size (N = 10) was another limitation, as it increases the risk of a type II error (null hypothesis accepted when it is false) and sampling bias. That said, our small sample size is similar to prior studies that investigate the impact of ROC on psychosocial outcomes, and was a pilot study aimed at highlighting associations of psychosocial outcomes with ROC participation. Future studies aimed at examining the impact of ROC on these outcomes should calculate sample size requirements as part of the study design. Additionally, our group of participants had attended ROC for a mean of 6.2 ± 2.7 years. Our participants’ high engagement with ROC, and the camp experiences of many of our participants, may be a well-established part of their regular annual routine. This may introduce bias into our results, as this cohort may be self-selecting. Future studies should aim to increase sample size and include participants with varying levels of involvement in ROC programming. Due to our small sample size, data stratification was not feasible; however, stratifying analyses by ROC participation and/or age ranges could help mitigate potential bias and allow for a more nuanced interpretation of findings within different developmental contexts.

The fatigue effect could also be a limitation of this study. Although the survey was brief, taking less than 10 min to complete, we were working with a pediatric population that was eager to return to their camp activities. This could have led to reduced attention while completing the survey and/or increased the likelihood that they would choose the same responses for each item toward the end of the survey [52]. Neville et al. (2019) reported that the use of questionnaires/surveys was less preferable for children attending ROC compared with qualitative approaches to psychosocial data collection including arts-based approaches and interviews [41]. Although the psychometric tools we used in this study were validated for use in pediatric/adolescent populations, these tools may be less engaging and more difficult to understand than other approaches [41]. Research may benefit from using a combination of psychometric tools and more in-depth analyses of the psychosocial health of childhood cancer patients such as child-centered arts-based approaches, which could reveal underlying thoughts and themes that may not be captured by quantifiable measures [53]. Additionally, collateral information from camp staff and/or caregivers may be included in the future to strengthen conclusions made from self-reported data. Another important consideration is the particular constructs we chose to evaluate in the current study. We evaluated hope, resilience, social support, and well-being, but other measures such as self-efficacy, self-esteem, and self-confidence may be impacted by the ROC experience and should be considered in future studies.

This study was also limited in racial diversity, as our participant population was largely Caucasian. Residential camps are common in North America, but Black and/or Indigenous families may feel uncomfortable sending their child to ROC due to a lack of cultural safety and the history of residential schooling in Canada [37,54,55]. As such, it is necessary to focus on diversifying the ROC environment, removing racial and cultural barriers to ROC participation, and investigating the impact of ROC on the psychosocial health of youth with lived cancer experience who have intersecting marginalities [37].

## 5. Conclusions

Levels of hope were significantly lower in childhood cancer patients three months post-ROC compared to scores while at ROC. These results suggest that the positive psychosocial outcomes that we observed during ROC may not be sustained once children return to their regular environments. Therefore, it may be beneficial to provide continued opportunities to promote psychosocial health for youth with lived cancer experience throughout the year so that they maintain their psychosocial health. Based on the results of this study, placing a greater focus on fall and winter ROC sessions may help to support the psychosocial health of youth with lived cancer experience throughout the year.

Overall, the results of this study suggest that while attending ROC, youth with lived cancer experience demonstrate high levels of resilience, hope, perceived social support, and overall mental well-being. These psychosocial outcomes could help youth with lived cancer experience cope with and adapt to the adversity brought upon them by cancer and treatment, while also providing them with resources to become well-adjusted throughout adulthood.

## Figures and Tables

**Table 1:** Descriptive statistics of study participants.

	All Participants *n* = 10
Mean Age (SD)	14.1 (2.5)
Median Age (Range)	13.5 (10–18)
Number of Participants on Active Treatment	1
**Gender (%)**	
Female	60.0%
Male	40.0%
Non-binary	0.0%
**Race/Ethnicity (%)**	
Southeast Asian	20.0%
Caucasian	50.0%
Chinese	30.0%
Asian/Hispanic	0.0%
Black/Caucasian	0.0%
**Diagnosis (%)**	
Leukemia	70.0%
Solid Tumour	20.0%
Brain Tumour	10.0%
Mean Number of Years Since Primary Cancer Diagnosis (SD)	7.6 (4.3)
Median Number of Years Since Primary Cancer Diagnosis	8
Mean Number of Years Attending Overnight ROC (SD)	4.7 (2.5)
Median Number of Years Attending Overnight ROC	6
Mean Number of Years Attending Any ROC (SD)	6.2 (2.7)
Median Number of Years Attending Any ROC	7.5

SD: standard deviation; ROC: recreational oncology camp.

**Table 2:** Mean scores for the CHS, CYRM, SPS-5, and SWEMWBS for participants (N = 10) at baseline, post-camp 1, and post-camp 2 with reference ranges and scoring thresholds, and effect sizes compared to previous timepoint [20,23,29,30].

	Baseline	Post-Camp 1	Post-Camp 2
Mean: CHS	28.30 ± 5.89	29.30 ± 6.72	23.70 ± 7.36
Reference Ranges: CHS	High (25–36)	High (25–36)	Moderate (19–24)
Effect Size: CHS		Small (d = −0.16)	Large (d = 0.80)
Mean: CYRM	65.40 ± 10.08	64.50 ± 8.38	61.20 ± 7.02
Reference Ranges: CYRM	High (63–67)	High (63–67)	Moderate (56–62)
Effect Size: CYRM		Small (d = 0.10)	Small (d = 0.43)
Mean: SPS-5	17.90 ± 2.78	18.30 ± 1.89	15.70 ± 4.17
Reference Ranges: SPS-5	High (15–20)	High (15–20)	High (15–20)
Effect Size: SPS-5		Small (d = −0.17)	Large (d = 0.80)
Mean: SWEMWBS	27.00 ± 3.71	27.60 ± 2.88	24.60 ± 4.58
Reference Ranges: SWEMWBS	>75th percentile (27–35)	>75th percentile (27–35)	<75th percentile (<27)
Effect Size: SWEMWBS		Small (d = −0.18)	Moderate (d = 0.79)

CHS: Children’s Hope Scale; CYRM: Child and Youth Resilience Measure; SPS-5: Social Provisions Scale-5; SWEMWBS: Short Warwick–Edinburgh Mental Wellbeing Scale; d: Cohen’s d.

## Data Availability

The raw data supporting the conclusions of this article will be made available by the authors upon request.
